# Wooded Semi-Natural Habitats Complement Permanent Grasslands in Supporting Wild Bee Diversity in Agricultural Landscapes

**DOI:** 10.3390/insects11110812

**Published:** 2020-11-18

**Authors:** Justine Rivers-Moore, Emilie Andrieu, Aude Vialatte, Annie Ouin

**Affiliations:** 1 DYNAFOR, Université de Toulouse, INRAE, 31320 Castanet-Tolosan, France; emilie.andrieu@inrae.fr (E.A.); aude.vialatte@inrae.fr (A.V.); annie.ouin@toulouse-inp.fr (A.O.); 2 LTSER Zone Atelier « PYRÉNÉES GARONNE », 31320 Auzeville-Tolosane, France

**Keywords:** semi-natural habitats, wild bees, wooded habitats, permanent grasslands, bee communities, pollen, interaction network

## Abstract

**Simple Summary:**

Loss of semi-natural habitats in agricultural landscapes negatively affects wild bees. These pollinators are, however, very important in agricultural landscapes as they enable the pollination of crops and wild plants. The aim of this study was thus to understand the respective roles of different wooded and herbaceous habitats in their ability to support a diversity of wild bees. We first found that wild bee communities differed between wooded and herbaceous habitats, some bee species being found in one type of habitat and not in the other. We also showed that wooded semi-natural habitats provide some species of pollen preferred by the bees. Finally, we found that in wooded habitats there are some interactions between plant and bee species that do not happen in permanent grasslands. However, the latter also plays an important role in the diversity of bees and plants, and these wooded and herbaceous habitats complement each other. Overall, our results underline the importance of maintaining a diversity of semi-natural habitats in agricultural landscapes to maintain a diversity of wild bees and thus promote the pollination of wild plants and crops.

**Abstract:**

Loss of semi-natural habitats (SNH) in agricultural landscapes affects wild bees, often negatively. However, how bee communities respond varies and is still unclear. To date, few studies have used precise descriptors to understand these effects. Our aim was to understand the respective and complementary influences of different wooded and herbaceous habitats on wild bee communities. We selected thirty 500-m radius landscapes on a gradient of a percentage of wooded SNH in south-western France. At each landscape, we sampled wild bees in spring 2016 and plants in spring 2015 and 2016 at the forest edge, in a hedgerow, and in a permanent grassland. Pollen carried by the most abundant bee species was collected and identified. Using beta diversity indices, we showed that wild bee community composition differs between the three SNH types, and especially between herbaceous and wooded SNH. Based on Jacobs’ selection index, we showed that pollen of some plant species recorded in wooded SNH are preferentially selected by wild bees. Studying the impact of the loss of each SNH type on the global bee-pollen interaction network, we found that wooded SNH contributed to its resilience, enabling specific plant–bee interactions. Overall, our results underline the non-negligible contribution of wooded SNH to the diversity of wild bees in agricultural landscapes, and thus the importance of maintaining different types of SNH.

## 1. Introduction

Since the 1960s, agricultural intensification has led to landscape simplification (removal of semi-natural habitats and enlargement of fields), which is one of the main causes of biodiversity decline, including the diversity and abundance of pollinators [1,2]. These practices not only have direct effects on pollinators, but also impact plant communities and floral resource availability, which themselves have strong effects on species richness and abundance of pollinators [3,4,5]. Approximately 75% of food crop species worldwide depend on animal pollination [6], and pollinators increase the yields of several crop species [7,8]. Wild bees, which pollinate a diversity of wild plant species, also contribute to crop pollination [9], often to a greater extent than managed honeybees [10,11,12]. As a consequence, they can compensate for possible yield losses caused by the increasing loss of honeybee colonies [2,13,14].

Loss of semi-natural habitats (SNH) in agricultural landscapes affects both wild bee diversity and pollination [15,16,17]. SNH provide food and nesting sites for wild bees [18], the decline of which may be more specifically due to loss of suitable host plants and lack of resources [19]. However, the amount of SNH in a landscape affects wild bees differently depending on the landscape context [3,20]. The term “semi-natural habitat” refers to different types of habitats (herbaceous, wooded) with no reference to their spatial configuration, and thus, their effect on bee diversity can vary widely. For instance, Winfree et al. [13] found a negative effect of the proportion of natural habitat (mainly forest) on wild bee abundance and species richness. Some studies have also shown that bee diversity decreases with an increase in the area of predominantly natural habitat [3]. In the end, only a few studies use precise descriptors to understand the impacts of SNH on pollinators [21], yet this information is needed for better farm management strategies to increase pollination potential in agroecosystems [22].

Among the different SNH present in agricultural landscapes, permanent grasslands and wooded habitats like hedgerows or small forests are the most common. The favorable role of grasslands in supporting wild bees and providing them with the resources they need is well known (see, e.g., [23,24]), whereas the contribution of wooded habitats has received little attention despite the fact they provide floral and nesting resources to bees. Hedgerow and forest edge’s flora comprise woody and herbaceous strata, and sometimes a high density of flowering bushes [25] that are scarcer in other landscape elements and are important for attracting bees. Hedges appear to be attractive habitats for wild bees [25,26], and their role is especially important in early summer, when the species richness of plants is higher than in other SNH [27]. Some trees and bushes in wooded habitats have also been shown to offer a more abundant and sugar-rich nectar than plant species found in permanent grasslands [28]. Although in some instances this seems to depend on local resources [29], several studies have found a higher bee diversity in wooded habitats than in adjacent habitats [30,31]. All these scattered studies have shown the importance that these wooded habitats can have for wild bees, and this link needs to be studied further, especially in a landscape context or on a larger scale.

Indeed, some studies have shown that bee diversity is associated with diverse habitats or resources in a landscape [32,33,34], as many species require several—and sometimes specific—habitats to maintain their population [18]. Because some of the wild and solitary bee species are specialist flower foragers, they may be more abundant in semi-natural features, such as grasslands and grassy strips, and look for more specific flowers than crops can provide [4]. Different habitats can provide a variety of resources that a species requires at a given time or at different stages of its life cycle [27,35,36,37]. Wooded habitats, which are less common than grasslands in many landscapes, play an important role in this complementarity [27].

Because bees need plant resources, and more than 75% of plants depend on pollinators to maintain their populations [4], plant–bee interactions are essential in agricultural systems. These links between species have been overlooked but, in the last years, the study of plant-pollinator networks to better understand biodiversity has expanded [38]. These networks describe the mutualistic interactions between species, and help the understanding of ecosystem stability and functions such as pollination [1,39]. The stability of these networks in the ecosystem depends, among others, on the number of species and the number of interactions between species, and can be affected by many environmental factors, loss of habitat being an important one [40,41,42]. Few studies have so far focused on plant–bee networks in the face of habitat loss, and particularly on the contribution of different types of SNH to this network on a bigger scale. It is, however, important to understand these processes in order to improve the stability and resilience of plant-pollinator networks in agricultural landscapes, and thus the pollination of wild and cultivated plants present in these ecosystems.

Following this greater goal of understanding how to improve pollinator diversity and pollination in agricultural landscapes, the aim of our study was to understand the respective and complementary influences of different wooded and herbaceous habitats on wild bee communities. To that end, we pursued three specific objectives: (1) To compare bee and plant communities in hedgerows, forest edges and permanent grasslands. Our hypothesis being that these communities differ, i.e., that wooded habitats host specific plant or bee species that are not found in grasslands; (2) to determine the proportion of pollen loaded by bees originating from wooded versus herbaceous SNH. Our hypothesis being that wild bees consume more wooded SNH plant species; (3) to study the impact of the loss of the three types of SNH on the plant-pollinator network at the scale of our study site. Our hypothesis being that wooded semi-natural habitats enable specific plant–bee interactions and contribute to the resilience of the network.

## 2. Materials and Methods

### 2.1. Study Site and Sampling Design

**Figure 1:**
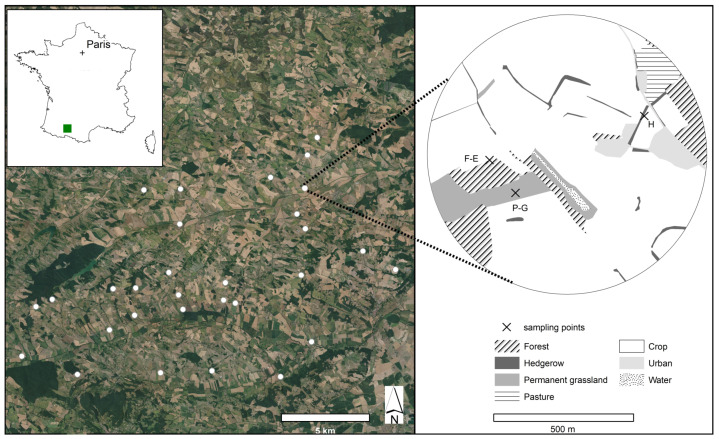
Maps showing the study area with on the left the 30 landscapes sampled, and on the right the location of the three sampling points in one of the 30 landscapes. P-G: permanent grassland, H: hedgerow and F-E: forest edge. BD ORTHO^®^ 20 cm—2019.

The study was conducted in the *Vallées et Coteaux de Gascogne* in south-western France (Figure 1), which is part of the Long-Term Socio-Ecological Research site ZA PYGAR ($43^{{}^{\circ}}$17′ N, $0^{{}^{\circ}}$54′ E). This hilly region (250–400 m a.s.l.) is characterized by a mosaic of grasslands, small forests and crop fields (mainly winter cereals) [43]. The climate is sub-Atlantic with slight Mediterranean influences (mean annual temperature, 12.5 ${}^{{}^{\circ}}$C; mean annual precipitation, 750 mm).

Based on French agricultural land cover data (Registre Parcellaire Graphique, RPG) and woodland cover data (BD TOPO^®^, IGN), we selected in this 220-km${}^{2}$ study site 30 circles with a radius of 500 m (hereafter referred to as landscapes). They were selected to maximize variations in the proportion of wooded semi-natural habitats cover (5–39%). In each landscape, we selected three sampling points of three SNH types: a forest edge, a hedgerow, and a permanent grassland (Figure 1). A forest edge is defined here as the interface between a forest and a cultivated field (annual crop or permanent grassland). A hedgerow is defined here as a line of trees between two crops. Both forest edges and hedgerows had to have a relatively constant orientation, to be as straight as possible and to be at least 100 m long. Hedgerows with at least a tree stratum were chosen. To avoid an effect of orientation, we avoided choosing south or north-facing forest edges and hedgerows, as these appeared to be used differently by insects and plants than other orientations [44,45]. A permanent grassland is defined here as an area dominated by herbaceous species that had not been sown or plowed for at least five years. Because seven of the 30 landscapes did not contain all three types of SNH, we finally sampled 30 hedgerows, 29 grasslands and 24 forest edges.

### 2.2. Data collection

Wild bees (Apoidae) were collected between May 20th and June 21st 2016 using an insect net along transects in each sampling point. Even if using only sweep nets to study bee communities may bias the outcome of the results [46,47], the use of pan traps to complement the sampling would not have allowed us to work on pollen and interaction networks. The net method was thus chosen to enable the individual storage of each bee and to avoid pollen transfer between specimens. Bees were hunted for 10 min, excluding time of capture and preparation of each individual insect. The transects were 100 m long and 5 m wide and were located in the middle of grasslands and along forest edges and hedgerows. To avoid an edge effect, the transects were at least 10 m from a border [29]. Sampling was carried out between 9:30 a.m. and 5:00 p.m., at a temperature of more than 17 ${}^{{}^{\circ}}$C, but the temperature could drop to 13 ${}^{{}^{\circ}}$C in sunny, windless, and cloudless conditions [48]. Each captured wild bee was placed individually in a vial containing paper soaked in ethyl acetate. It was then mounted and identified to species level by two specialists in wild bee identification, based on their bee reference collection and on taxonomic literature [49,50]. We did not catch honeybees *Apis mellifera* because the abundance of this managed species is likely to be related to beekeeping rather than to a direct effect of landscape structure. After identification, the females of the most abundant species only (>4 individuals in the whole dataset) were kept for pollen sampling, because it would not have been possible to differentiate between the effect of the specific diet of rare species on the pollens they harvest versus a habitat effect. Pollen grains were collected on the bee’s body with a fine tweezer and a moistened brush and were deposited directly into a droplet of water placed on a microscope slide. Pollen grains from each individual bee were observed under a ×400 optical microscope and identified to genera or species level based on the pollen reference collection of the *INRAe du Magneraud* [51,52].

Botanical surveys were conducted in May and June 2016 in forest edges and hedgerows. Concerning permanent grasslands, 13 of them were surveyed in April and May 2015 and 16 in May and June 2016. We considered that botanical composition in permanent grasslands is constant from year to year under similar management [53]. The surveys comprised abundance-dominance records of all vascular plant species according to the Braun-Blanquet scale [54] in three vegetation layers (herbaceous: 0–1 m, shrubs: 1–3 m and trees: >3m). Data were collected along a 25-m transect on each side of the hedgerows, on a 50-m transect along the forest edges, and wandering freely in grasslands.

### 2.3. Data Analysis

All statistical analyses were performed with R 3.6.3 [55].

Because light or seasons have been shown to sometimes impact bee presence or diversity (see, e.g., [56]), we first and foremost tested the impact of weather conditions and the date of capture on the abundance and diversity of bees, using Wilcoxon tests for quantitative variables and Kruskal–Wallis tests for categorical variables.

#### 2.3.1. Abundance and Diversity Analyses

Individual-based species accumulation curves were created to compare total species richness and abundance between hedgerows, forest edges and permanent grasslands. We aggregated bee data within each type of SNH and calculated accumulation curves with the package *iNEXT* [57]. Following MacGregor-Fors and Payton [58], we used 84% confidence intervals to determine statistical significance between accumulation curves with an error rate of 0.05.

We evaluated differences in wild bee abundance and species richness between sampling points in hedgerows, forest edges and grasslands with Kruskal–Wallis rank tests followed by Dunn’s test of multiple comparisons (package *dunn.test* [59]). Dunn’s tests *p*-values were adjusted following Bonferroni’s method to account for multiple comparisons.

We compared bee community composition in the three types of SNH using beta diversity. The same method was used for communities of botanical species. Based on the work of Baselga [60], we divided beta diversity into two components: nestedness (species subset) [61] and spatial turnover (species replacement) [62]. Using the *betapart* package [63], one by one, we calculated total dissimilarity between the three types of SNH with Sørensen’s index $\beta_{sor}$. Nestedness ($\beta_{sne}$) and turnover ($\beta_{sim}$/Simpson dissimilarity) are part of $\beta_{sor}$ and can be found as follows:

$$\beta_{sor}\;=\;\beta_{sim}+\beta_{sne}\equiv \frac{b+c}{2a+b+c}\;=\;\frac{b}{b+a}+\left(\frac{c-b}{2a+b+c}\right)+\frac{a}{a+b}$$

where *a* is the number of common species shared by two sites, *b* is the number of species unique to the poorest site, and *c* the number of species unique to the richest site. Only presence/absence data were used for these calculations. To test for the difference between beta diversity values expected by chance (neutral sampling effect) and the differences driven by the habitat type filter, we used null model controls [64,65] constructed with random permutations of samples.

Bee community composition was also compared between the sampling points within each type of SNH. To that end, we calculated Sørensen’s index $\beta_{sor}$ between each pair of sampling points within one type of SNH, and used Kruskal–Wallis rank tests followed by Dunn’s test of multiple comparisons to identify significant differences between the three types of SNH. The *dunn.test* [59] and *betapart* [63] packages were used for these analyses.

#### 2.3.2. Pollen Availability and Preference

For the sake of convenience, hereafter we use the term plant species for a plant species resulting from botanical surveys and pollen species for the name of a plant species determined from the pollen grains collected on a bee’s body. Because of the high variability of pollen size and quantity among plant species [66] and because of the different foraging efficiency between bee species [42], we chose to use presence–absence data for pollens and for botanical data. This choice may have biased our results, but as suggested by Vialatte et al. [52], the use of presence–absence data appears to be the least-biased way to estimate pollen availability at a study site and pollen carrying by bees. The pollen availability index (PA) is a proxy of the availability of pollen species in a given study site. The PA for a given plant species is the number of times a species occurs compared to the total number of samples (a sample being a plant species found at a given sampling point) in the study site. Only plant species that were found on the wild bees we captured were included in the analyses, i.e., the species that actually supply the pollen to the bees. The list of pollen species allowed us to calculate a pollen harvest rate (HR) at the species level, which is the number of times a pollen species was present at least once on a bee compared to the total number of pollen samples found. To allow for the use of HR and PA for the same plant/pollen species, and because some of the pollen species were not determined at the species level, we had to switch some species in the botanical data to the level of genus.

According to Jacobs [67], selective feeding occurs when a feeder uses co-occurring resources at different rates. We applied Jacobs’s selection index D to evaluate wild bees’ preferences for a given pollen species, using:

$$D_{i}\;=\;\frac{HR_{i}-PA_{i}}{HR_{i}+PA_{i}-2HR_{i}xPA_{i}}$$

where $HR_{i}$ is the consumption rate of pollen of species i (i = 1–49) and $PA_{i}$ is the availability of pollen species i in the sampling points (N = 83). A positive Jacobs’s selection index indicates bees have a higher preference for a given pollen species (here called “over-selected” species) than would be expected from the resource abundance, while a negative index indicates a lower consumption (here called “under-selected” species). Using the bootstrap method, we conducted random resampling of the data concerning the pollen consumption rate, using 5000 bootstrap samples to calculate the confidence intervals (2.5 and 97.5%) of D for each plant species.

Hereafter, when we use the term “specific” to an SNH type, we mean that, in our dataset, the species was found only in this type of SNH.

#### 2.3.3. Network Analysis

We constructed a pollen–bee interaction network, at the level of our study site, based on the interactions observed between bee species and pollen species in each type of habitat. To that end, we used a subset of the whole dataset that only included the most abundant bee species (>4 ind.). Based on a study by Evans et al. [40], the importance of each SNH type for the robustness of this network was examined by calculating the impact of the removal of pollen/bee interactions observed in a given habitat type on the network [40]. Because of the differences in sample sizes, we resampled sampling points in each habitat type using the bootstrap method (random sample with no replacement) to reach N = 24 in each type of habitat. We constructed 5000 interaction networks, each containing data from the 24 forest edges, and from 24 grasslands and 24 hedgerows randomly selected among the original 29 grasslands and 30 hedgerows. In each of the 5000 networks, we measured the percentage of remnant interactions and species in the network resulting from the removal of pollen and bee species in each type of SNH. The mean value of remnant species and interactions was then calculated for each type of SNH.

## 3. Results

Wilcoxon tests between wild bee abundance or species richness and luminosity, wind speed, clouds and temperature gave all *p*-values < 0.01. Kruskall–Wallis tests between bee diversity variables and date of sampling gave *p*-values > 0.05. It means none of the weather or date variables had a significant influence on bee diversity.

### 3.1. Bee Communities within and between SNH Types

**Figure 2:**
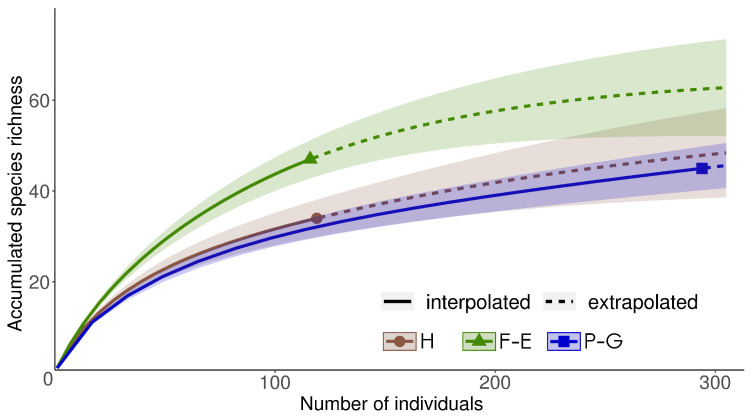
Individual-based randomized species accumulation curves comparing wild bee species richness between the three types of habitat (H: hedgerows, F-E: forest edges and P-G: permanent grasslands). The shaded areas represent 84% confidence intervals. Non-overlapping of these intervals indicates significant differences with an error rate of 0.05.

In total, we netted 529 individual wild bees belonging to 77 species (see Appendix A for a complete list of species). The most abundant were the social species *Lasioglossum malachurum* (N = 72), *Bombus lapidarius* (N = 31) and *Lasioglossum villosulum* (N = 29) and the solitary species *Eucera nigrifacies* (N = 46) and *Halictus simplex* (N = 24). We collected 294 individuals belonging to 45 species in grasslands, 116 individuals belonging to 47 species in forest edges and 119 individuals belonging to 34 species in hedgerows. Accumulation curves between the three types of SNH show the significantly higher total species richness of wild bees in forest edge habitats compared to grasslands, despite its weaker abundance of bees (Figure 2). In forest edges, 75% (N = 35) of the species were represented by one or two individuals. This was the case of 53% (N = 18) of the species in hedgerows and 46% (N = 21) in grasslands. Specific species, i.e., found only in one type of SNH, accounted for 30% (N = 14) in forest edges, 24% (N = 8) in hedgerows, and 33% (N = 15) in grasslands.

**Figure 3:**
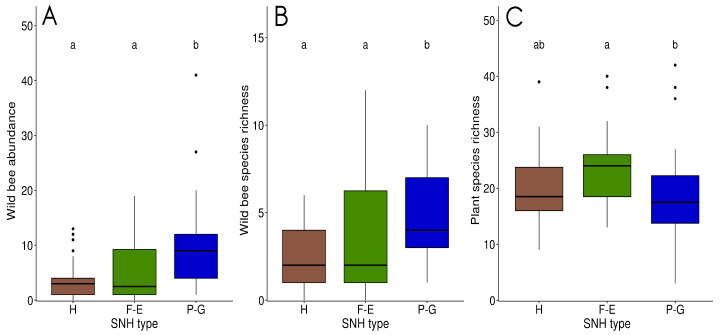
Bee abundance (**A**) and species richness (**B**), and plant species richness (**C**) in the sampling points of each semi-natural habitats (SNH) type (H: hedgerows, F-E: forest edges and P-G: permanent grasslands). The letters above each plot indicate significant differences between land cover types after multiple comparisons (Dunn’s test) following the Kruskal–Wallis (KW) test. Boxplots represent the median value, first and third quartile.

Per sampling point, bee abundance and species richness differed significantly among the three types of SNH (respectively KW = 17, *p* < 0.001 and KW = 13.4, *p* < 0.01). Grasslands had significantly greater abundance (median = 9, SE = 1.56) and species richness (median = 4, SE = 0.51) than hedgerows (respectively 3±0.69 and 2±0.31; median ± SE) and forest edges (respectively 2.5±1.08 and 2±0.51; median ± SE) (Figure 3).

**Figure 4:**
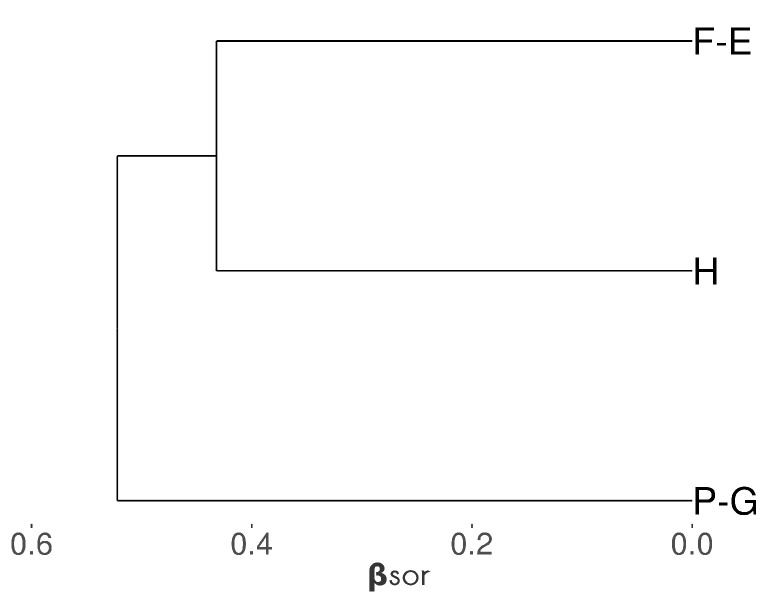
Dendrogram of the three types of SNH (H: hedgerows, F-E: forest edges and P-G: permanent grasslands) ranked according to Sørensen’s index ($\beta_{sor}$) calculated between the three wild bee communities. $\beta_{sor}$ is lower between H and F-E than between P-G and the two wooded SNH, indicating a lower difference in species composition in these communities.

Wild bee beta diversity (inter SNH-type) between hedgerows and forest edges was 0.43 for $\beta_{sor}$ ($\beta_{sim}$ = 0.32, $\beta_{nes}$ = 0.11). It was 0.54 (0.47, 0.07) between grasslands and hedgerows, and 0.5 (0.49, 0.01) between grasslands and forest edges. The difference in composition between grasslands and the two wooded SNH was greater than the difference between hedgerows and forest edges (Figure 4), and was more due to species turnover than to community nestedness.

**Figure 5:**
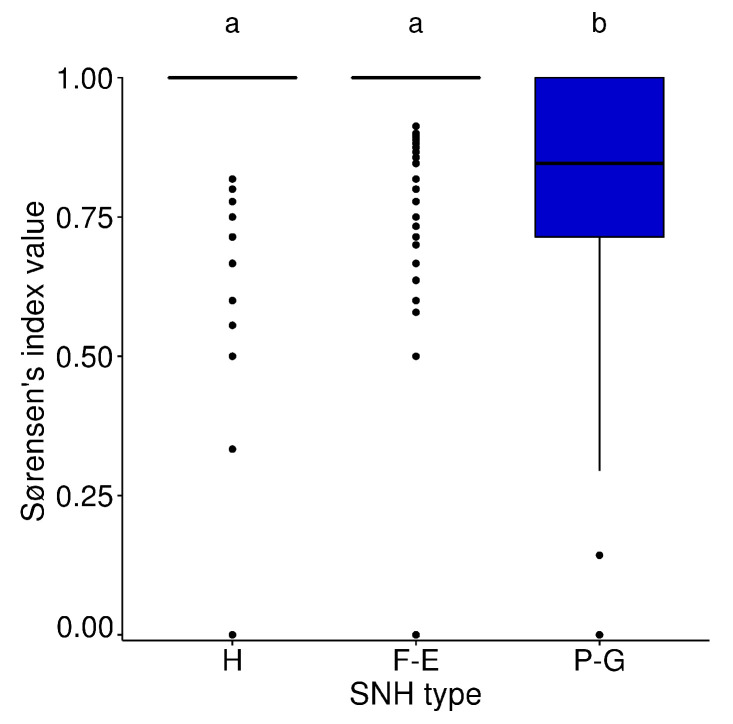
Boxplots of Sørensen’s index between sampling points of each type of SNH (H: hedgerows, F-E: forest edges and P-G: permanent grasslands). The letters above each plot indicate significant differences between types of SNH after multiple comparisons (Dunn’s test) following the KW test. Boxplots represent median value, first and third quartile.

The median of the $\beta_{sor}$ values between sampling points (intra SNH-type) in hedgerows and forest edges was similar (median = 1.0, σ=0.22 for these two types of SNH, whereas in permanent grasslands it was 0.83 (σ=0.23). Beta diversity was significantly higher in each of the two wooded SNH than in permanent grasslands (KW = 292, *p*-value < 0.001) (Figure 5).

### 3.2. Plant Communities within and between SNH Types

A total of 308 plant species were observed in the three types of SNH. A total of 73.7% (N = 227) were considered “entomophilous” (i.e., pollen and/or nectar harvested by insects) and were used in the study. The most common were small ligneous species: *Rubus* spp. (observed in 53 sampling points/83), *Prunus spinosa* L. (47/83) and *Hedera helix* L. (43/83). When all the sampling points were pooled, forest edges had the highest plant species richness (144 species), followed by grasslands (119) and last hedgerows (113). Per sampling point, plant species richness differed significantly among grasslands and forest edges (KW = 7, *p* < 0.05), but not between hedgerows and the two other types of SNH. Forest edges had significantly higher species richness (median = 24, σ=6.85) than grasslands (17.5±8.77). The species richness of hedgerows (18.5±6.47) did not differ significantly from that of the two others (Figure 3). Plant beta diversity between grasslands and hedgerows was 0.58 for $\beta_{sor}$ ($\beta_{sim}$= 0.56, $\beta_{nes}$ = 0.02). It was 0.51 (0.46, 0.05) between grasslands and forest edges, and 0.31 (0.28, 0.03) between hedgerows and forest edges.

### 3.3. Pollen Selection by Wild Bees

**Figure 6:**
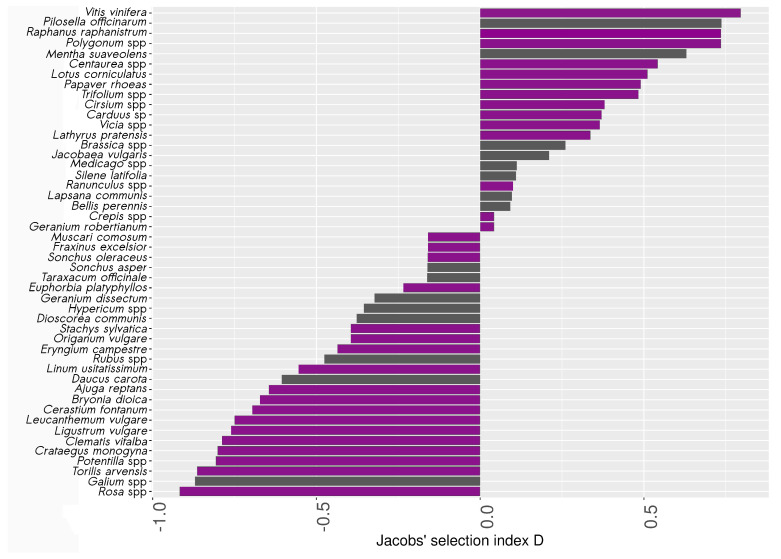
Selectivity of pollen sources by wild bees (Jacobs’ index D). An index > 0 indicates higher relative abundance of the pollen species on bees’ bodies compared to its availability in the botanical survey, whereas an index < 0 indicates negative selection of the species. Purple bars represent species with a significant positive or negative D, following the calculation of the confidence intervals (2.5 and 97.5%) with bootstrap resampling in pollen species, and grey otherwise. For this analysis, some pollen species have been switched at the genus level.

Among the 349 bees belonging to the 27 most abundant species retained for pollen analysis, we found 462 samples of pollen belonging to 55 species (see Appendix B for a complete list of species). A total of 15% of the bees carried no pollen. A maximum of six pollen species were found per individual, but 95% of the bees carried only one or two species. Pollen harvest rates (HR) ranged from 0.003 to 0.106 (mean = 0.02, σ=0.027). The five most frequently harvested species or genera were *Lotus corniculatus* L., *Lathyrus pratensis* L., *Trifolium repens* L., *Cirsium* spp. and *Ranunculus* spp. Seven pollen species were not found in the vegetation surveys and were thus not used to calculate the Jacobs’ index. Five of these seven species (*Arabidopsis thaliana* (L.) Heynh, *Lathyrus aphaca* L., *Linum perenne* L., *Lonicera caprifolium* L. and *Sinapis arvensis* L.) were found on only one bee, one on two individuals in the same sampling place (*Solanum nigrum* L.), and *Tilia ${}^{x}$europaea* L. was found on 12 bees at different locations. Jacobs’ selection index D was therefore calculated for only 48 of the 55 species. The index went from −0.91 to 0.81 (mean = −0.09, σ=0.51) (Figure 6). A total of 46% of the species had a positive Jacobs’ index. Most of the species with a high D had a low pollen availability index (PA) and a higher HR. For instance, *Vitis vinifera* L., the species with the highest Jacobs’ index, was encountered only three times in the botanical surveys (PA = 0.001), but each time was found on bees in these samples. It was highly harvested compared to its estimated availability in the study site, and is therefore considered here as « over-selected » by wild bees. Among the pollen species « over-selected » by bees (i.e., with a significant positive Jacobs’ index), 31% were found only in hedgerows or in both forest edges and hedgerows, like *Vitis vinifera* L., *Raphanus raphanistrum* L. or *Papaver rhoeas* L. Eight percent were specific to grasslands (e.g., *Polygonum* spp.), and 54% were found in both herbaceous and wooded habitats (e.g., *Lotus corniculatus* L.).

### 3.4. Bee–Pollen Interaction Network

**Figure 7:**
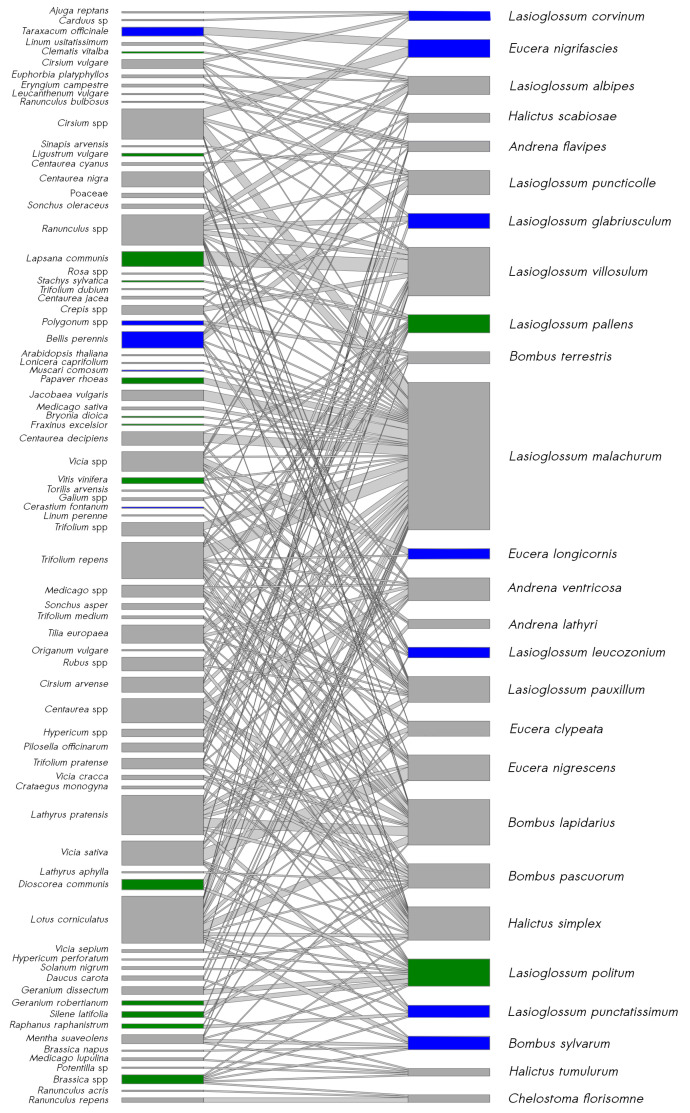
Bipartite graph of the bee–pollen interaction network containing the most abundant species in this study (>4 individuals). The width of the link indicates the frequency of the interaction. Blue bars represent species found only in grasslands; green bars represent species that were found in wooded SNH (i.e., hedgerows and/or forest edges).

**Figure 8:**
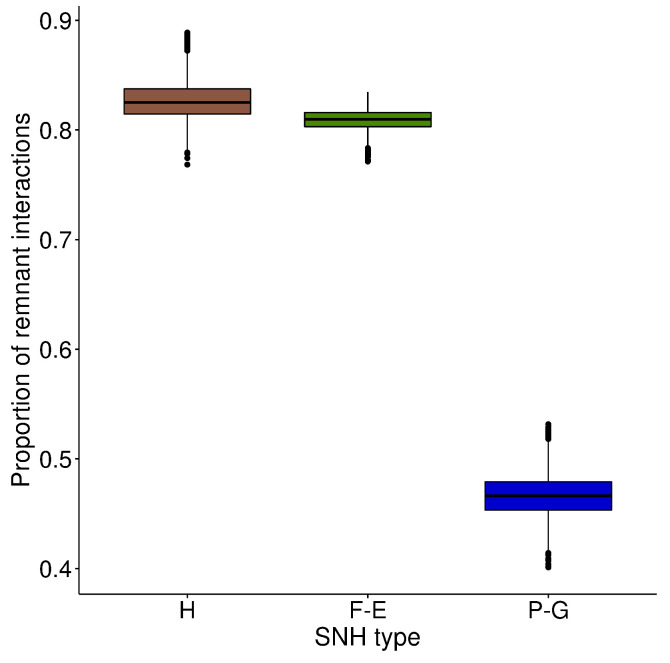
Impact of the removal of species of each type of SNH (H: hedgerows, F-E: forest edges and P-G: permanent grasslands) on the proportion of bee–pollen interactions remaining in the network. Boxplots represent median value, first and third quartile. Mean is equal to median.

The overall study-site network, constructed with a subset of data from the most abundant bee species, comprised 226 unique interactions between 27 bee species and 55 pollen species (Figure 7). All the bee species were linked to at least two plant species and the median number of links per bee species was seven. The eusocial bee species *Lasioglossum malachurum* had the maximum number of links, with 38 interactions with plant species. In total, 37% of the bee species were specific to grasslands, i.e., caught only in this type of SNH, and 7% were specific to wooded habitats. The removal of all grasslands from the network resulted in the mean reduction of 53.3% (σ=1.9) of species interactions (Figure 8), 36.8% (σ=2.8) of plant species and 38.8% (σ=2.1) of bee species. Removal of hedgerows led to a mean reduction of 17.4% (σ=1.7), 9.8% (σ=1.9) and 0.1% (σ=0.6) in species interactions, plant species and bee species, respectively. Removal of forest edges resulted in a mean loss of 19.1% (σ=1.0), 10.1% (σ=1.6) and 0.8% (σ=1.4) in species interactions, plant species and bee species, respectively. Removing the two wooded habitats had less impact than removing only grasslands on bee and plant species richness of the network. However, together, wooded habitats hosted about 40% of unique interactions (Figure 8), i.e., links between bee and plant species not present in the other habitats.

## 4. Discussion

The data highlight the important role played by permanent grasslands for wild bees in agricultural landscapes. However, it also highlights the important complementary role of two other habitats, hedgerows and forest edges, for wild bee diversity. We showed that hedgerows and forest edges host specific bee and plant species, some of which pollen was over-selected (i.e., highly harvested compared to its availability in the study site) by wild bees. Moreover, these wooded semi-natural habitats support specific plant–bee interactions that are not observed in grasslands. Below we discuss the complementary contribution of the two wooded semi-natural habitats to permanent grasslands for bees in agricultural landscapes.

### 4.1. Wooded Habitats Have Specific Bee and Plant Communities

In our study, bee and plant community composition partially differed between the three types of semi-natural habitat, due to species turnover, i.e., a change in species composition between habitat types. Nestedness was particularly low between grasslands and forest edges, meaning few species are shared by these two SNH. These findings validate our first hypothesis: communities differ in the types of semi-natural habitat, and bee and plant communities in wooded habitats are not a simple subset of communities found in grasslands. Consequently, the presence of a wooded SNH in a landscape increases gamma diversity, i.e., the total species diversity at the landscape scale [68]. Furthermore, it seems that communities in wooded SNH, particularly in forest edges, are more varied than communities in grasslands that show lower beta diversity, at least in this season. Beta diversity is the component of regional diversity that accumulates as a result of compositional differences between local species assemblages [69]. In this part of the study, we considered local assemblages as each of the communities found in sampling points, and regional assemblage as the total community of a type of SNH. A high beta diversity between species assemblages of wooded SNH sampling points means then that there is a large difference of species composition between the sampling points in these types of SNH. Forest edges are ecotones, special ecosystems in which two habitats and their communities interact [70]. They thus have more chance of differing from each other than permanent grasslands. Their higher variability suggests that adding a wooded element to a landscape would result in more species diversity to landscape scale biodiversity than a grassland, which may have a lower intrinsic value. All these results are consistent with those obtained by Bartual et al. [21], who concluded that biodiversity patterns vary across SNH types and across subsites within SNH types. The higher diversity between sampling points at forest edges could also be due to differences in their management that were not taken into account in our study. Impacting local floral resources affects bee communities [71,72], and management of these SNH could play a role in enhancing bee or plant biodiversity [73,74,75].

Extending this study to the entire flight period of the bees could have enabled a more precise understanding of the role of wooded habitats to support bee and plant communities. The different species of bees are not present at the same times in agricultural landscapes, and it seems that they adapt and move according to the resources present at different periods [37]. Some studies are already beginning to show the importance of hedges and forest edges in providing resources over extended periods for wild pollinators, especially at times when few resources are present in cultivated plots (see, e.g., [76]). However, this research is still in progress, and our study over a very short period of time already shows the value of wooded SNH.

### 4.2. Wooded Habitats Provide Specific and Over-Selected Floral Resources

Some of the pollen species harvested by wild bees were found to be more frequently selected than others, compared to their availability in the study site. Even if, according to Montoya et al. [39], abundant plant species are more likely to receive the attention of pollinators, other factors can play a role in bees’ foraging choices. Their visits to plant species may vary depending on bees and flower traits. For instance, nectar composition but also flower shape or color can play a role in attraction of visiting bees [42]. Moreover, some specialist bees can be more selective and have a preference for specific plant families or genera [77]. Among the pollen species over-selected by bees according to Jacobs’ index, less than 10% were specific to grasslands, whereas a much larger proportion (31%) came from plants we observed only in wooded SNH. This could be because, as we found, grasslands have a smaller intra-SNH-type beta diversity. Species found in a permanent grassland are therefore more likely to be found in several other grasslands, and thus to have a high pollen availability index and a negative Jacobs’ index. On their side, plant communities in wooded SNH are more diverse and resemble each other less, given the higher intra-SNH-type beta diversity index. The pollen species harvested by wild bees in these types of SNH may thus be less frequent in the total study site, have a smaller pollen availability index and hence a positive Jacobs’ index. It shows the importance of wooded SNH in providing wild bees with diverse and attractive resources, and pollens that they preferentially look for. Among the significantly over-selected pollen species found only on bees caught in wooded SNH, two were mainly found in wooded or shaded habitats in our study region: *Geranium robertianum* L. [78] and *Vitis vinifera* L. The latter probably originates from former grape vines that have become rewilded, and today only survive in wooded SNH. Even if *Vitis vinifera* is mainly self or wind pollinated, other authors have shown that it was frequently visited by bees, who harvest the partly sterile pollen from hermaphrodite flowers [79,80]. The two other species with a positive Jacobs’ index that were found only on bees collected in wooded SNH, *Raphanus raphanistrum* L. and *Papaver rhoeas* L., are weeds mainly found in crop fields and on their borders. They may thus be found at the base of hedgerows or forest edges, but not in permanent grasslands. As we did not sample plants growing in crop fields, in our study, weed species had a weak pollen availability index, which consequently influenced Jacobs’ index. To limit the effect of rare species in network analysis, we only analyzed pollen carried by the most abundant bee species. This resulted in a pollen data subset that was much more limited in the two wooded SNH. Indeed, these SNH hosted lots of uncommon bee species. Here we showed wild bees’ preference for certain pollen species specific to wooded SNH, but the positive role of these habitats is underestimated, as lots of bee and hence pollen species were lacking in the subset.

### 4.3. Wooded Habitats Participate in the Diversity of Interactions in Plant–Bee Networks

The network we studied resembles other networks described in the literature, with the majority of species connected with few other species, and a few species being highly connected [1,42]. Even if only the most abundant species were taken into account, our results confirmed our third hypothesis concerning the positive contribution of wooded SNH to the resilience of plant–bee networks by allowing for specific interactions. While only the loss of grasslands resulted in a major disappearance of bee and plant species, the removal of wooded habitats from the network impacted the number of interactions by causing the loss of about 40% of unique plant and bee links in the network. In our study, permanent grasslands enabled almost double the number of single wild bee–pollen interactions. The importance of permanent grasslands for wild bees is widely reported in the literature [81,82,83], but their positive role has been little studied in connection with interaction networks. In a study led by Evans and collaborators in 2013, the most important habitat for the robustness of a complex network of ecological networks to habitat loss was mature hedgerows. Grassland was the least important habitat [40]. The results obtained by these authors differed from our study, but confirm the positive role played by wooded habitats in increasing the number of species interactions in our network. Ecological interactions can lead to the loss of ecological functions that determine the dynamics of populations, communities, and ecosystems [84]. These trophic links between species reinforce network complexity [39] and its stability and resilience to environmental change [41]. Wooded habitats thus play a fundamental role in supporting bee–plant networks and the stability of agricultural ecosystems.

### 4.4. Management of SNH in Agricultural Landscapes

As we showed in this study, wooded and herbaceous semi-natural habitats complement each other and all participate in a greater diversity of bees in agricultural landscapes. Their difference in botanical composition may be one of the main reasons explaining their respective roles to provide floral but also nidification resources to a diversity of bees [21,85]. Understanding beta-diversity between bee and plant communities of these different habitats is a first step and will help protect regional bee diversity [69]. Because management of wooded habitats is a probable source of diversity and because grasslands may have a lower biodiversity threshold, management practices at landscape and SNH scales should be taken into account in projects aimed at maintaining biodiversity in agricultural landscapes [74,86]. According to [87], heterogeneity at the landscape and local scales is a key to sustain biodiversity in farmlands. At a time when many insects are disappearing and crop pollination is a central issue in agriculture, wooded habitats like hedgerows and small forests should not be seen as limits to agricultural practices but rather as assets to be taken into account in overall management, for a more agro-ecological farming.

## 5. Conclusions

Taken together, our data suggest that wooded SNH like forest edges or hedgerows contribute to bee diversity and complement grasslands in supporting diverse and functionally efficient bee communities in rural landscapes. Wooded SNH promote bee diversity in agricultural landscapes by hosting specific bee and plant communities, providing pollen, and enabling plant–bee interactions that do not occur in permanent grasslands. This result underlines the importance of maintaining diverse SNH in agricultural landscapes, and of including wooded habitats in management and conservation projects, not only to maintain bee and other pollinators diversity (see, e.g., [88]) but also to enable efficient pollination of a wide range of crops and wild plants.

## Data Availability

Data is available here: https://doi.org/10.15454/PA6UHY; https://doi.org/10.15454/X8G2LI; https://doi.org/10.15454/SBLX2C.

## Appendix A. Complete List of Bee Species

**bee species**	
*Andrena agilissima*	*Eucera nigrescens*
*Andrena alfkenella*	*Eucera nigrifacies*
*Andrena carantonica*	*Eucera numida*
*Andrena cineraria*	*Eucera taurica*
*Andrena dorsata*	*Halictus scabiosae*
*Andrena flavipes*	*Halictus simplex*
*Andrena florea*	*Halictus smaragdulus*
*Andrena fulvago*	*Halictus subauratus*
*Andrena haemorrhoa*	*Halictus tumulorum*
*Andrena impunctata*	*Hylaeus brevicornis*
*Andrena labialis*	*Hylaeus communis*
*Andrena labiata*	*Hylaeus gibbus*
*Andrena lathyri*	*Lasioglossum albipes*
*Andrena minutula*	*Lasioglossum corvinum*
*Andrena nigroaenea*	*Lasioglossum discum*
*Andrena ovatula*	*Lasioglossum glabriusculum*
*Andrena ranunculi*	*Lasioglossum interruptum*
*Andrena simontornyella*	*Lasioglossum laevigatum*
*Andrena strohmella*	*Lasioglossum lativentre*
*Andrena subopaca*	*Lasioglossum leucozonium*
*Andrena variabilis*	*Lasioglossum malachurum*
*Andrena ventricosa*	*Lasioglossum medinai*
*Andrena vetula*	*Lasioglossum morio*
*Andrena viridescens*	*Lasioglossum pallens*
*Anthidium manicatum*	*Lasioglossum pauxillum*
*Anthophora plumipes*	*Lasioglossum politum*
*Bombus hortorum*	*Lasioglossum punctatissimum*
*Bombus lapidarius*	*Lasioglossum puncticolle*
*Bombus lucorum*	*Lasioglossum villosulum*
*Bombus pascuorum*	*Lasioglossum zonulum*
*Bombus pomorum*	*Nomada basalis*
*Bombus pratorum*	*Nomada kohli*
*Bombus sylvarum*	*Nomada striata*
*Bombus terrestris*	*Osmia bicornis*
*Ceratina cucurbitina*	*Osmia brevicornis*
*Ceratina cyanea*	*Osmia caerulescens*
*Chelostoma florisomne*	*Osmia niveata*
*Eucera clypeata*	*Sphecodes gibbus*
*Eucera longicornis*	

## Appendix B. Complete List of Pollen Species and Genera

**pollen species**	
*Ajuga reptans* L.	*Lotus corniculatus* L.
*Arabidopsis thaliana* (L.) Heynh.	*Medicago lupulina* L.
*Bellis perennis* L.	*Medicago sativa* L.
*Brassica napus* L.	*Medicago* spp
*Brassica* spp	*Mentha suaveolens* Ehrh.
*Bryonia dioica* Jacq.	*Muscari comosum* (L.) Mill.
*Carduus* sp	*Origanum vulgare* L.
*Centaurea cyanus* L.	*Papaver rhoeas* L.
*Centaurea decipiens* Thuill.	*Pilosella officinarum* Vaill.
*Centaurea jacea* L.	*Polygonum* spp
*Centaurea nigra* L.	*Potentilla* spp
*Centaurea* spp	*Ranunculus acris* L.
*Cerastium fontanum* Baumg.	*Ranunculus bulbosus* L.
*Cirsium arvense* (L.) Scop.	*Ranunculus repens* L.
*Cirsium* spp	*Ranunculus* spp
*Cirsium vulgare* (Savi) Ten.	*Raphanus raphanistrum* L.
*Clematis vitalba* L.	*Rosa* spp
*Crataegus monogyna* Jacq.	*Rubus* spp
*Crepis* spp	*Silene latifolia* Poir.
*Daucus carota* L.	*Sinapis arvensis* L.
*Dioscorea communis* (L.) Caddick & Wilkin	*Solanum nigrum* L.
*Eryngium campestre* L.	*Sonchus asper* (L.) Hill
*Euphorbia platyphyllos* L.	*Sonchus oleraceus* L.
*Fraxinus excelsior* L.	*Stachys sylvatica* L.
*Galium* spp	*Taraxacum officinale* (L.) Weber
*Geranium dissectum* L.	*Tilia ${}^{x}$europaea* L.
*Geranium robertianum* L.	*Torilis arvensis* (Huds.) Link
*Hypericum perforatum* L.	*Trifolium dubium* Sibth.
*Hypericum* spp	*Trifolium medium* L.
*Jacobaea vulgaris* Gaertn.	*Trifolium pratense* L.
*Lapsana communis* L.	*Trifolium repens* L.
*Lathyrus aphaca* L.	*Trifolium* spp
*Lathyrus pratensis* L.	*Vicia cracca* L.
*Leucanthemum vulgare* Lam.	*Vicia sativa* L.
*Ligustrum vulgare* L.	*Vicia sepium* L.
*Linum perenne* L.	*Vicia* spp
*Linum usitatissimum* L.	*Vitis vinifera* L.
*Lonicera caprifolium* L.	
